# Ancestral protein reconstruction reveals the mechanism of substrate specificity in FN3K-mediated deglycation

**DOI:** 10.1038/s42003-026-09967-3

**Published:** 2026-04-03

**Authors:** Jenet K. Matlack, Robert E. Miner, Jameela Lokhandwala, Jennifer M. Binning

**Affiliations:** 1 Department of Molecular Medicine, Morsani College of Medicine, University of South Florida, Tampa, FL USA; 2 Department of Molecular Oncology, H. Lee Moffitt Cancer Center and Research Institute, Tampa, FL USA; 3 Cancer Biology PhD Program, University of South Florida, Tampa, FL USA

**Keywords:** Post-translational modifications, Kinases

## Abstract

Protein glycation is a detrimental byproduct of carbohydrate metabolism, and nearly all organisms encode kinases that facilitate the removal of early glycation products. In humans, these repair functions are performed by Fructosamine-3 and Ketosamine-3 kinases (FN3K, KT3K). Recent structural studies identified conserved residues essential for FN3K activity, but the molecular basis for substrate discrimination between FN3K and KT3K remains unknown. Here, we show that substrate-binding residues are highly conserved and do not confer specificity in the FN3K family. Using APR, we resurrected four ancestral fructosamine kinases that recapitulate the distinct substrate preferences of FN3K and KT3K. Through mutational studies and structural analysis, we identify an evolutionarily tuned allosteric network that modulates substrate selection through long-range intramolecular interactions. Our findings define the mechanism of substrate selectivity within the FN3K family and establish a framework for the development of selective FN3K inhibitors.

## Abstract (web summary)

Ancestral protein reconstruction of fructosamine kinase family reveals dynamic evolutionarily tuned allosteric network that modulates substrate selection through long-range intramolecular interactions in human FN3K.

## Introduction

Protein glycation is a non-enzymatic post-translational modification that is implicated in many metabolic and aging-related disorders, such as diabetes, cardiovascular disease, neurodegenerative disease, and cancer^1–6^. Protein glycation occurs when reducing sugars, including glucose and ribose, react with free amine groups on amino acids to form an intermediate Schiff base. These Schiff bases can rearrange to form early glycation products called ketosamines. If left unchecked, glycation can undergo further rearrangements into advanced glycation end products (AGEs). Through receptors for advanced glycation end products (RAGEs), AGEs are activators of the NF-ΚB inflammatory signaling pathway, promoting both cellular damage and apoptosis^7^. While AGEs are thought to be irreversible products^8,9^, early glycation products can be enzymatically repaired via Fructosamine-3 kinases (FN3Ks).

FN3Ks are conserved across the tree of life and are present in both eukaryotic and prokaryotic organisms^10^. FN3Ks repair glycated proteins by phosphorylating the O3’ of the sugar moiety on glycated amines. This generates an unstable product that reverts to the Schiff base intermediate, ultimately generating the unmodified amine, an inorganic phosphate, and byproducts such as 3-deoxyglucosone. There are two paralogues of FN3Ks in higher orders of life: FN3K and Ketosamine-3 kinase (KT3K or FN3K-RP). In humans, these enzymes share 64% sequence identity at the amino acid level^11,12^. Despite this similarity, KT3Ks and FN3Ks recognize different glycated substrates. While both enzymes share overlapping activity on ribulosamines, only FN3K is capable of phosphorylating fructosamines^12,13^. This diversification of FN3K substrates later in evolution highlights the evolutionary necessity of fructosamine repair in higher orders of life^13^.

Human FN3K (HsFN3K) has recently emerged as a therapeutic target due to its role in deglycating Nuclear factor erythroid 2–related factor 2 (NRF2), a master regulator of oxidative stress. Under normal physiological conditions, NRF2 is tightly regulated by Kelch-like ECH-associated protein 1 (KEAP1) and acts as a transcription factor in the presence of reactive oxygen species. In many cancers, NRF2 is constitutively activated, leading to metabolic reprogramming, increased proliferation, and resistance to therapy^14,15^. Depletion of FN3K reduced tumor burden in NRF2-driven hepatocellular carcinoma and non-small cell lung cancer xenograft mouse models, implicating FN3K as a cellular regulator of NRF2 function^16^. Additionally, HsFN3K can directly phosphorylate ribose-glycated NRF2 peptide, further supporting NRF2 as a physiological substrate for FN3K^17^. Despite FN3K’s emerging relevance in cancer biology, the extent to which FN3K and KT3K have redundant or distinct physiological roles remains unclear. This presents a critical challenge, as it is unknown whether dual inhibition of both KT3K and FN3K would be detrimental to viability, or if selective targeting may enable a therapeutic window for FN3K antagonists. Thus, a deeper understanding of the structural and evolutionary mechanisms underlying FN3K and KT3K substrate specificity is essential for guiding rational drug design.

Recently, we and others reported the structural basis for deglycation by HsFN3K^17,18^. These studies defined a set of conserved substrate-binding residues essential for catalysis on 1-deoxy-1-morpholino-D-fructose (DMF), a small molecule fructosamine mimic. While these studies advance our understanding of FN3K protein deglycation, they did not address the distinct yet overlapping substrate specificities of FN3K and KT3K. The structural and evolutionary basis for this functional divergence remains unresolved. In this study, we address this gap by integrating phylogenetic analysis, ancestral protein reconstruction (APR), and mutational biochemistry to elucidate the molecular determinants of substrate specificity within the fructosamine kinase family. We show that substrate-binding residues in HsFN3K are conserved across all fructosamine kinases and are required for the phosphorylation of both fructosamines and ketosamines. Using APR, we reconstructed ancestral enzymes that recapitulate the substrate preferences of HsFN3K and HsKT3K, despite differing by only 12 amino acids. Through systematic mutagenesis, we identified that substrate specificity in FN3Ks is regulated not by direct interactions within the active site, but by an allosteric network that governs fructosamine recognition through long-range intramolecular communication. By defining how FN3Ks evolved to selectively recognize glucose-glycated proteins, we lay the foundation for structure-guided therapeutic targeting of this clinically relevant enzyme.

## Results

### Identification of FN3K residues required for glycated protein recognition

**Fig. 1:**
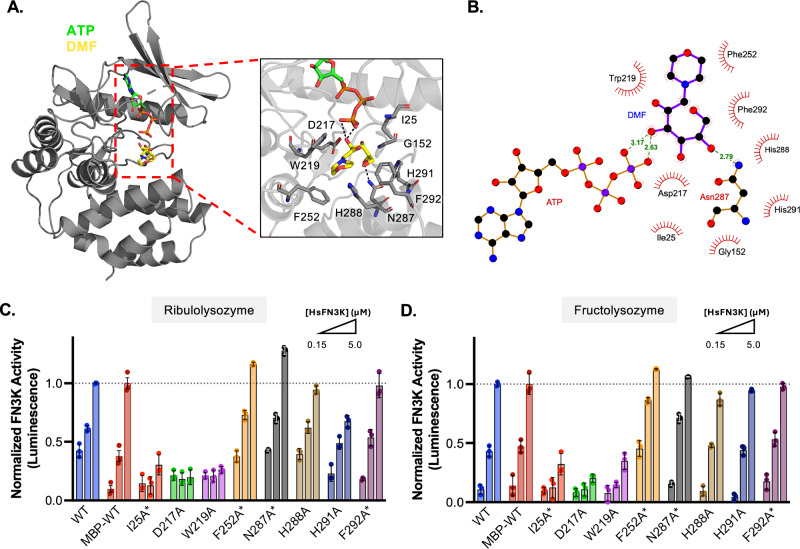
Substrate-binding residues in HsFN3K are required for small molecule and protein substrates. **A** Ribbon diagram of HsFN3K bound to DMF and ATP (PDB: 9CXM). The substrate-binding pocket is highlighted in red. Dashed lines in the inlet represent hydrogen bonding. **B** Ligand-protein interaction analysis of HsFN3K with DMF and ATP. **C**, **D** Kinase assay of HsFN3K WT, I25A*, D217A, W219A, F252A*, N287A*, H288A, H291A, and F292A* on ribulolysozyme and fructolysozyme. The represented concentrations are 0.15 µM, 1.25 µM, and 5.0 µM. ATP consumption was measured by luminescence. Each data point represents the means of technical triplicates; error bars indicate standard error. Astericks (*) represents proteins that contain an MBP-tag.

Recent high-resolution structural studies have identified key residues within HsFN3K that mediate binding to the substrate-mimetic DMF (Fig. 1A). Site-directed mutagenesis confirmed the functional importance of W219, H288, and H291 in binding DMF and substrate recognition^17,18^. To identify additional residues contributing to substrate engagement across the FN3K family, we performed a ligand-protein interaction analysis using the HsFN3K-ATP-DMF pre-catalytic complex structure. This analysis revealed nine residues within the substrate-binding pocket that contact DMF: I25, D217, W219, F252, N287, H288, H291, F292, and G152 (Fig. 1B). Among these residues, N287 stabilizes the sugar moiety through hydrogen bonding, allowing D217 to function as the catalytic base. The side chains of W219, F252, H288, H291, and F292 form stabilizing π-stacking interactions with the substrate, while G152 contributes through backbone interactions.

To evaluate the functional relevance of these contacts, each residue was individually mutated to alanine, and kinase activity was assessed using a luciferase-based assay. Unlike previous studies, which primarily utilized DMF, we used fructosamine- and ribulosamine-modified lysozyme to assess FN3K activity against glycated proteins (Fig. 1C, D, Supplementary Fig. 1A–C). Select HsFN3K mutants retained an MBP-tag to enhance stability. Their activities were normalized to the matching tagged or untagged HsFN3K wild type to ensure consistent comparisons. We observed that only I25A, D217A, and W219A lost activity towards both fructo- and ribulolysozymes. We previously showed that D217A and W219A retain ATP binding but fail to interact with DMF^18^. Although I25 is solvent-exposed in the apo structure, it reorients to the catalytic pocket in the ternary complex, such that it is positioned at the interface between ATP and DMF (Supplementary Fig. 1D). Using differential scanning fluorimetry (DSF), we qualitatively assessed the ability of HsFN3K I25A to bind ATP (Supplementary Fig. 1E). The I25A mutant fails to exhibit ATP-induced thermal stabilization, suggesting a role for I25 in positioning both ATP and substrate for catalysis.

Our data also established that HsFN3K F252A, N287A, H288A, H291A, and F292A mutants retain activity towards both glycated lysozymes. Consistent with our previous study, which showed that HsFN3K H288A impaired but did not abolish kinase activity towards DMF, this mutation showed a slight decrease in activity towards fructolysozyme. Interestingly, H288A did not impair activity towards ribulolysozyme. We observed an opposing effect for H291A, which did not impair activity towards fructolysozyme but showed a decrease in activity towards ribulolysozyme. Additionally, we observed that F252A and N287A mutants enhance activity towards both substrates (Supplementary Fig. 1B-C).

Together, these data define the HsFN3K residues critical for substrate binding and HsFN3K activity. Of the nine residues that contact DMF, only I25, D217, and W219 are essential for catalytic activity, suggesting that these residues function as universal substrate recognition elements within the FN3K catalytic pocket.

### Conserved substrate-binding residues do not account for differences in substrate specificity between FN3Ks and KT3Ks

**Fig. 2:**
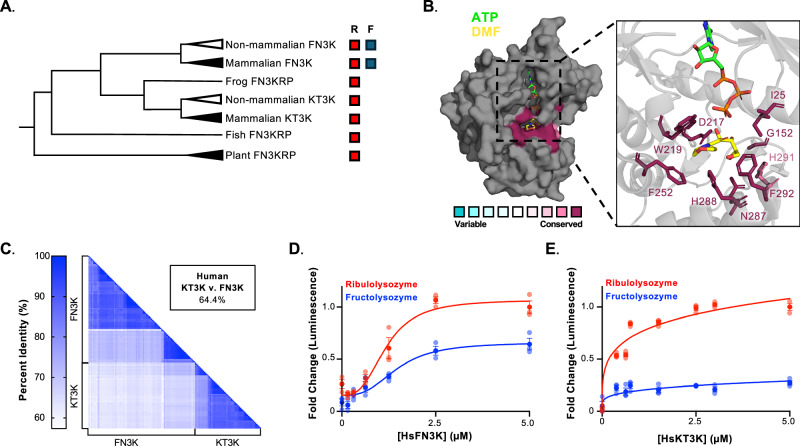
Substrate specificity differs between FN3K and KT3K despite conservation of substrate-binding residues. **A** Unrooted phylogeny tree of fructosamine kinase family. Substrate specificity towards ribulosamine (R) and fructosamine (F) is indicated on the right of the phylogeny tree. **B** Conservation analysis of fructosamine kinase family represented on HsFN3K bound to DMF and ATP (PDB: 9CXM). The colors indicate the level of conservation of residues according to the scale. Substrate-binding residues are highlighted in the inlet. **C** Percent identity matrix of amino acid sequences of FN3Ks and KT3Ks post-duplication. Colors are represented by the scale bar. Kinase assay of HsFN3K (**D**) and HsKT3K (**E**) on ribulolysozyme (red) and fructolysozyme (blue). Curves were normalized to luminescence at 5 µM against ribulolysozyme. The enzyme was titrated in the indicated concentration range, and ATP consumption was measured by luminescence. Each data point represents the means of technical triplicates; error bars indicate standard error.

Previous phylogenetic and biochemical analyses have shown that mammalian and non-mammalian FN3Ks phosphorylate fructosamines, whereas KT3Ks display a narrower substrate preference (Fig. 2A)^12,13^. Conservation analysis across >14,000 fructosamine kinase family members revealed that all nine substrate-binding residues identified in the HsFN3K-DMF co-structure are highly conserved across the family (Fig. 2B). This conservation persists in both FN3Ks and KT3Ks, despite their overlapping yet distinct substrate specificities. Of note, H291 has a slightly higher degree of variability, but remains highly conserved in mammalian and non-mammalian KT3Ks (86% conserved, Supplementary Fig. 2A, B). We next compared global sequence identities within and between the two subfamilies. While FN3Ks and KT3Ks exhibit high sequence conservation within each lineage, percent identity drops significantly when comparing members across the two clades (Fig. 2C).

To experimentally validate differences in substrate preference, we expressed and purified recombinant HsFN3K and HsKT3K and tested their activity using a luciferase-based kinase assay. As expected, HsFN3K phosphorylated fructolysozyme (Fig. 2D), whereas HsKT3K exhibited no activity toward this substrate (Fig. 2E). Both enzymes retained activity on ribulolysozyme, a common fructosamine kinase substrate. Given that this assay measures the conversion of ATP to ADP, we also performed direct substrate phosphorylation assays. HsFN3K was able to phosphorylate fructolysozyme; however, no substrate phosphorylation was observed for HsKT3K (Supplementary Fig. 2C).

These data demonstrate that the amino acids directly involved in substrate binding are conserved across the fructosamine kinase family, and therefore do not account for the divergent substrate specificities observed between FN3Ks and KT3Ks. These findings suggest that additional residues, likely acting outside the substrate-binding pocket, underlie the evolutionary diversification in substrate preference.

### Ancestral enzymes recapitulate origins of FN3K substrate specificity

**Fig. 3:**
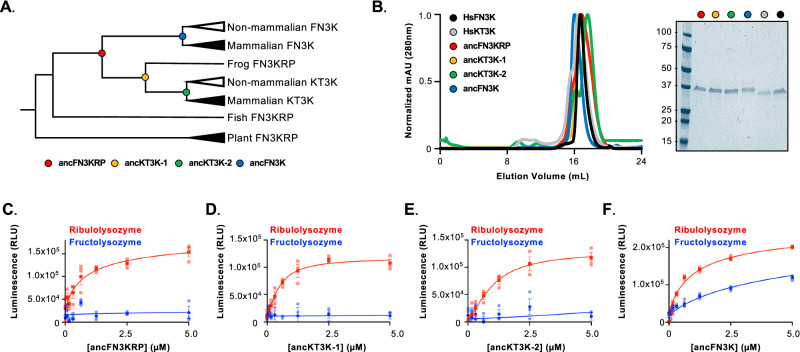
Ancestral reconstruction of fructosamine-3 kinase family recapitulates substrate specificity. **A** Unrooted phylogeny tree of fructosamine kinase family with locations of identified ancestral proteins. ancFN3KRP (red), ancKT3K-1 (yellow), ancKT3K-2 (green), and ancFN3K (blue) are represented as colored circles on the tree. **B** Size-exclusion chromatography profile of HsFN3K, HsKT3K, ancFN3KRP, ancKT3K-1, ancKT3K-2, and ancFN3K. The inset shows the Coomassie-stained SDS-PAGE of each protein. Kinase assay of ancFN3KRP (**C**), ancKT3K-1 (**D**), ancKT3K-2 (**E**), and ancFN3K (**F**) on ribulolysozyme (red) and fructolysozyme (blue). The enzyme was titrated in the indicated concentration range, and ATP consumption was measured by luminescence. Each data point represents the means of technical triplicates; error bars indicate standard error.

To identify the evolutionary adaptations underlying FN3K and KT3K substrate specificity, we employed ancestral protein reconstruction (APR) to resurrect ancestral fructosamine kinases that recapitulate the substrate preferences of human FN3K and KT3K. This approach integrates information from multiple sequence alignments and phylogenetic trees to infer the most probable ancestral sequences at key evolutionary nodes of divergence in the tree^19,20^. Given that FN3K emerged after fish speciation, sequences from bacterial orthologs were excluded, and phylogenetic analysis began with plant orthologs^12,13^. The resulting phylogenetic tree was evaluated by comparison with previous phylogenetic analyses of the FN3K family and the Ensembl Compara Gene Trees framework for FN3K^12^. From this, we inferred the most probable sequences of four ancestral proteins of interest (Fig. 3A; Supplementary Fig. 3): ancFN3KRP, which predates the gene duplication event; ancKT3K-1 and ancKT3K-2, both from the KT3K lineage; and ancFN3K, which lies on the FN3K lineage preceding the emergence of HsFN3K. All four ancestral sequences preserve the nine substrate-contacting residues identified in Fig. 1B (Supplementary Fig. 5A).

Each ancestral protein was recombinantly expressed and purified, eluting as a monodisperse peak by size-exclusion chromatography at a similar elution volume to HsFN3K and HsKT3K (Fig. 3B). Thermal stability analysis revealed that ancKT3K-1 and ancKT3K-2 displayed equivalent or increased melting temperatures compared to HsKT3K. In contrast, ancFN3K had a 6 °C lower melting temperature compared to HsFN3K (Supplementary Fig. 5B, C). Across all reconstructed ancestral sequences, the mean posterior probability was high (0.95–0.96), including at all the functionally important residues. Therefore, the change in thermostability observed is unlikely to arise from a canonical “consensus effect” which is tied to uncertainty (Supplementary Fig. 4A–D)^21^.

We next assessed the catalytic activity of these reconstructed proteins using glycated lysozymes as substrates. All four ancestral proteins retained activity towards ribulolysozyme, but only ancFN3K exhibited activity towards fructolysozyme (Fig. 3C–F). These results demonstrate that the reconstructed ancestral enzymes recapitulate substrate preferences observed in extant KT3Ks and FN3Ks, and that the ability to repair fructosamines may have emerged specifically within the FN3K lineage.

### Reconstructing the neofunctionalization of fructosamine phosphorylation in FN3Ks

**Fig. 4:**
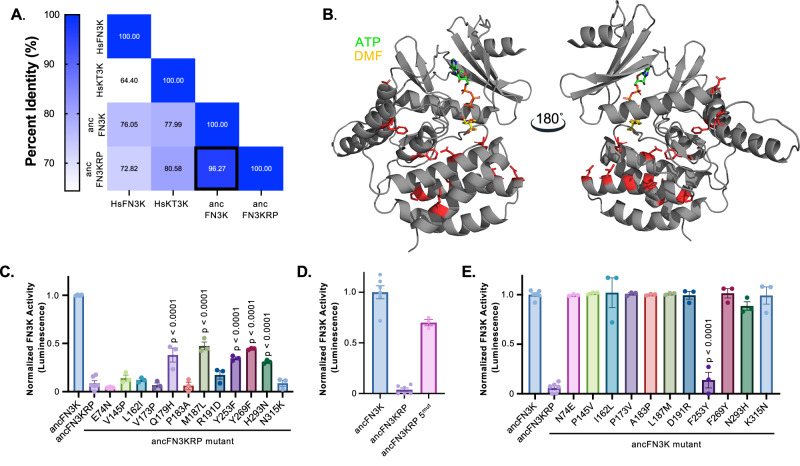
Systematic mutagenesis of ancFN3KRP and ancFN3K. **A** Percent identity matrix of HsFN3K, HsKT3K, ancFN3KRP, and ancFN3K. **B** AlphaFold3 model of ancFN3K. DMF and ATP were modeled into the structure by aligning HsFN3K-ATP-DMF with ancFN3K in PyMOL. The 12 amino acid residues that differ between ancFN3K and ancFN3KRP are highlighted in red as sticks. Kinase assay of ancFN3KRP gain-of-function (**C**, **D**) and ancFN3K loss-of-function (**E**) mutations against DMF. Each bar graph represents the activity at 5 µM of enzyme. Each bar represents means of technical triplicates, except for ancFN3KRP WT and ancFN3K WT, where *n* = 6; error bars indicate standard error. *P*-value was determined using Dunnett’s multiple comparisons test.

To identify the molecular determinants that underlie the substrate specificity differences between FN3Ks and KT3Ks, we focused on two reconstructed ancestral enzymes: ancFN3KRP and ancFN3K. These two ancestral proteins recapitulate the substrate preferences observed in their extant counterparts despite differing at only 12 amino acid positions (Fig. 4A; posterior probabilities listed in Supplementary Fig. 6B). All 12 residues are located on the periphery of the substrate-binding pocket (Fig. 4B), providing a focused framework to dissect the molecular basis of substrate specificity between FN3K and KT3K.

To elucidate the evolutionary changes that enabled deglycation of fructosamines, we conducted reciprocal mutagenesis to functionally convert ancFN3KRP into ancFN3K (gain-of-function) and ancFN3K into ancFN3KRP (loss-of-function) by introducing the 12 residue substitutions individually and in combination. Because early glycation adducts undergo chemical rearrangements over time, leading to increased heterogeneity, we assessed kinase activity using the model substrate, DMF, a well-established fructosamine mimetic. Among the 12 amino acid substitutions, five mutants (Q179H, M187L, Y253F, Y269F, and H293N) conferred enzymatic activity toward DMF when introduced into ancFN3KRP, indicating partial gain of FN3K-like specificity (Fig. 4C). While none of the single substitutions restored activity to the level of wild-type ancFN3K, combining all five substitutions (ancFN3KRP 5^mut^) further enhanced activity toward DMF, reaching approximately 70% of ancFN3K wild-type levels (Fig. 4D). Importantly, activity toward ribulolysozyme was retained for all gain-of-function mutations, establishing that these mutations do not broadly disrupt catalytic activity (Supplementary Fig. 6C, D).

We next performed complementary loss-of-function mutagenesis in the ancFN3K background to identify substitutions that would reduce or eliminate fructosamine activity, thereby rendering the enzyme more KT3K-like. Each of the 12 residues was individually reverted to its ancFN3KRP identity. In contrast to the gain-of-function results, only one substitution, F253Y, reduced activity towards DMF to a level comparable to WT ancFN3KRP (Fig. 4E), suggesting that F253 plays a central role in maintaining fructosamine specificity. Notably, the H179Q variant was insoluble in *E. coli* expression systems and could not be purified for activity assays. Together, these data demonstrate that substrate specificity divergence between FN3K and KT3K arose from a small number of amino acid substitutions following gene duplication. Among these, F253 (corresponding to HsFN3K F244) emerged as a key determinant in fructosamine recognition in the FN3K lineage.

### Substrate specificity in HsFN3K is modulated by peripheral residues identified in ancestral enzymes

**Fig. 5:**
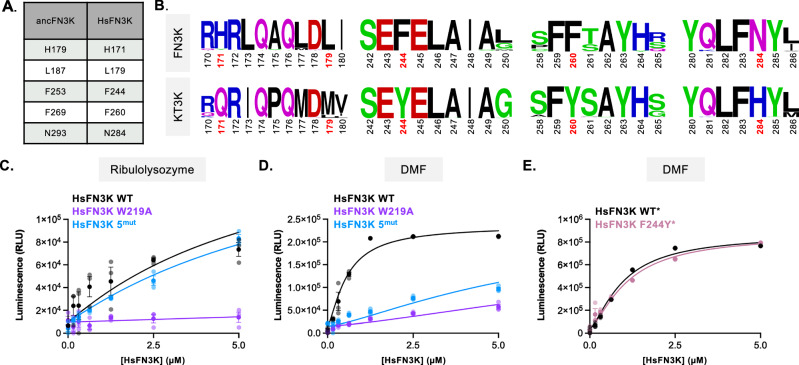
Identified residues in ancestral proteins cause loss of function in human FN3K. **A** Important positions of ancestral FN3K and their respective positions in human FN3K. **B** LogoPlot of FN3Ks and KT3Ks. HsFN3K 5^mut^ amino acid residues identified in ancestral proteins are highlighted in red. Kinase assay of HsFN3K WT, W219A, and 5^mut^ against ribulolysozyme (**C**) and DMF (**D**). **E** Kinase assay of HsFN3K WT* and HsFN3K F244Y*. Asterisks (*) indicates that the protein has an MBP-tag. Each data point represents the means of technical triplicates; error bars indicate standard error.

To investigate whether residues identified through ancestral protein reconstruction contribute to substrate specificity in HsFN3K (Fig. 5A; full table listed in Supplementary Fig. 6A), we first examined their conservation across the FN3K family. All five residues are highly conserved among both mammalian and non-mammalian FN3Ks and KT3Ks (Fig. 5B). Moreover, the residues surrounding these are also well-conserved, suggesting that substrate recognition is governed by a tightly constrained structural environment.

To determine whether these residues regulate substrate specificity in HsFN3K, we introduced the corresponding ancestral substitutions into HsFN3K to generate a five-point mutant, HsFN3K 5^mut^ (H171Q/M179L/F244Y/F260Y/N284H). We then assessed enzymatic activity. HsFN3K 5^mut^ retained activity towards ribulolysozyme (Fig. 5C), but lost activity toward fructosamines (Fig. 5D; Supplementary Fig. 7A). The combination of five mutations is sufficient to restrict activity to ribulosamines, recapitulating the narrower substrate preference characteristic of KT3K. Additionally, HsFN3K 5^mut^ exhibited an 8 °C increase in melting temperature relative to wild-type HsFN3K (Supplementary Fig. 7B), consistent with a more conformationally stable, KT3K-like state. Given that a single mutation (F253Y) in the ancestral enzyme was sufficient to restrict DMF activity, we next tested whether the analogous mutation in HsFN3K (F244Y) could independently recapitulate this effect. In contrast to the ancestral context, HsFN3K F244Y retained activity toward DMF (Fig. 5E), establishing that this mutation alone is insufficient to alter substrate specificity in the human enzyme. Together, these data demonstrate that multiple adaptations on the periphery of the substrate-binding pocket contribute collectively to substrate specificity in HsFN3K. These findings underscore the importance of cooperative interactions between distal residues in shaping functional divergence within the FN3K family.

### Intramolecular interaction analysis reveals dynamic allosteric regulation through residue 244 in FN3Ks and KT3Ks

**Fig. 6:**
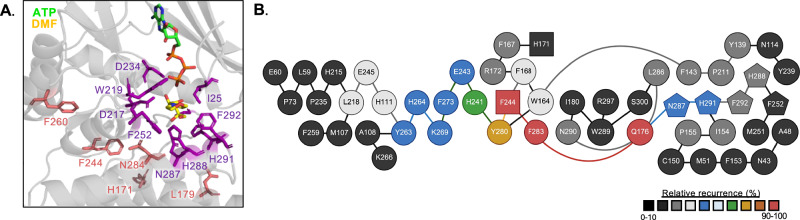
Protein structure network analysis of HsFN3K. **A** Substrate-binding pocket of HsFN3K-ATP-DMF structure. HsFN3K 5^mut^ residues are highlighted in salmon (e.g., F244). DMF binding residues are highlighted in purple (e.g., W219). **B** WebPSN network analysis of HsFN3K (PDB: 9CX8). Squares signify residues identified in this paper. Pentagons signify substrate-binding residues. Relative recurrence represents the strength of interactions and is indicated by the scale bar. Node colors are representative of the recurrence in network analysis and follow the same scale. Created in BioRender https://BioRender.com/0xc7owf.

Structural mapping of the HsFN3K 5^mut^ residues onto the ATP-DMF-bound HsFN3K structure revealed that none make direct contact with the substrate or substrate-binding residues (Fig. 6A). This suggests that these amino acids influence activity through indirect mechanisms, potentially via allosteric regulation of the active site. Comparison of the apo and substrate-bound HsFN3K structures showed no substantial conformational changes upon ligand binding (Supplementary Fig. 8A), indicating that the determinants of specificity may be pre-configured in the apo state.

To explore whether these residues participate in a secondary interaction network influencing the substrate-binding site, we examined residue-level interactions within a 6 Å cutoff. Residues 171, 179, 244, and 260 exhibited no contacts with substrate-binding residues in either HsFN3K or HsKT3K. Although residue 284 formed interactions with H288, N287, F252, and D217, key amino acids in the HsFN3K substrate-binding pocket, these interactions were conserved in both HsFN3K and HsKT3K and did not differ in distance or geometry, suggesting that residue 284 does not contribute to specificity through direct structural interactions (Supplementary Fig. 8B).

Given the well-established role of long-range, allosteric networks in kinase regulation^22,23^, we next performed Protein Structure Network (PSN) analysis to evaluate potential allosteric signaling within FN3K (Fig. 6B). Using a 3 Å distance cutoff, we identified the shortest communication pathways that pass through the HsFN3K 5^mut^ residues. HsFN3K F244 and H171 are predicted to form an interaction network that extends to five of the nine substrate-binding residues, forming a distinct communication pathway not found in KT3K-like proteins, such as *A. thaliana* FN3K (AtFN3K) (Supplementary Fig. 9). Together, these results support our mutational studies and provide a framework for how the five specificity-determining residues may function through dynamic allosteric networks rather than direct substrate engagement. In particular, residue 244 emerges as a central regulator of long-range intramolecular communication, facilitating a shift in allosteric connectivity that modulates substrate specificity in FN3Ks and KT3Ks.

## Discussion

Protein kinases represent one of the most therapeutically exploited classes of enzymes in the human proteome. FN3K has emerged as a therapeutic target due to its role in protein repair and regulation of redox signaling. Notably, FN3K has been implicated in the post-translational control of NRF2, a master regulator of oxidative stress responses and a known driver of chemoresistance in several cancers^24–27^. Given the long-standing challenges associated with developing NRF2 antagonists, there is a growing interest in identifying alternative therapeutic strategies for NRF2-driven cancers^28^. Recent studies have demonstrated that NRF2 is susceptible to non-enzymatic glycation and that FN3K-mediated deglycation can restore its transcriptional activity. These findings highlight FN3K as a potential vulnerability in NRF2-addicted tumors. However, to fully exploit FN3K as a therapeutic target, we need a detailed understanding of the molecular mechanisms underlying FN3K’s function, particularly its substrate specificity, which to date has remained poorly defined. Our study addresses this unresolved question and reveals that FN3K substrate specificity is not regulated within the active site itself, but instead arises from an allosteric network that modulates fructosamine discrimination.

In this study, we describe the molecular basis of substrate specificity in the fructosamine kinase family. FN3Ks and KT3Ks share overlapping activity on ribulosamines, yet only FN3K is capable of phosphorylating fructosamines. A central and unexpected finding of this work is the conservation of the FN3K active site. Using the recently published HsFN3K-ATP-DMF pre-catalytic structure, we identified nine residues that coordinate DMF, a fructosamine mimetic, within the active site of HsFN3K. All nine residues that coordinate DMF are highly conserved across the FN3K family, despite the clear functional divergence between FN3Ks and KT3Ks in their ability to phosphorylate fructosamines. This rules out a model in which substrate selectivity is mediated by direct recognition within the substrate-binding pocket. Instead, it suggests that molecular features distal to the active site must govern the ability of FN3Ks to repair fructosamines and ketosamines. Through a combination of structural analysis, ancestral protein reconstruction (APR), and biochemical characterization, we experimentally show that substrate specificity is not regulated by FN3K active-site residues, but instead evolved through minimal changes on the periphery of the catalytic core. Ancestral proteins reconstructed from before and after the FN3K-RP duplication event differ in sequence identity by only ~4%, yet they display distinct substrate preferences that closely mirror the extant FN3K and KT3K enzymes. Importantly, the acquisition of fructosamine activity in ancestral FN3Ks was accompanied by a loss of thermal stability (Supplementary Fig. 10). The ancFN3KRP 5^mut^, which gained the ability to deglycate fructosamines, resulted in a 16 °C decrease in melting temperature. This observation is consistent with prior studies showing that the evolution of new enzymatic functions is often accompanied by a loss of protein stability^29–32^. The substantially higher stability of HsFN3K relative to its ancestral counterpart suggests that subsequent compensatory mutations were required to buffer this destabilization, enabling fructosamines to become physiologically relevant substrates.

Our mutational analyses further reveal that FN3K specificity cannot be reduced to a single residue “switch.” Although a single substitution at position 253 was sufficient to toggle fructosamine activity in ancestral proteins, the analogous mutation in HsFN3K was not sufficient to alter substrate preference. Only when multiple amino acid substitutions were introduced simultaneously could HsFN3K (HsFN3K 5^mut^) be functionally restricted to ribulosamines, thereby recapitulating KT3K-like specificity. These results support a model in which substrate selectivity in FN3Ks is governed by a distributed network of residues that modulate catalysis through structural and dynamic effects, rather than by a single residue serving as the specificity “switch”.

Protein structure network analysis provides a mechanistic framework for mapping long-range intramolecular networks that regulate substrate specificity. Residues such as F244, which do not directly engage substrates, act as key nodes within intramolecular communication pathways that connect peripheral regions of the protein to the substrate-binding pocket. In HsFN3K, these pathways propagate through multiple residues to influence five of the nine substrate-coordinating positions, offering a structural explanation for how distal substitutions modulate catalytic outcomes without altering active-site composition. Together, our ancestral reconstruction and network analyses converge on a unified model in which FN3K substrate specificity is fine-tuned by allosteric coupling rather than direct substrate discrimination

These findings have important implications for drug discovery efforts targeting FN3K. The high conservation of the active site across FN3Ks and KT3Ks suggests that ATP-competitive or substrate-mimetic inhibitors will likely target both enzymes indiscriminately. This raises the possibility that FN3K inhibition could lead to off-target effects, particularly in tissues where KT3Ks may have independent functions. Additional studies that define the biological role of FN3K and KT3K will be critical to understand the therapeutic consequences of a pan-FN3K inhibitor. Moreover, our separation-of-function mutations provide powerful tools for dissecting the physiological roles of FN3K in vivo. Future studies can now ask whether fructosamine or ribulosamine repair is more critical in specific tissues or disease states. This will be especially valuable in models of metabolic dysfunction or NRF2-driven cancers. Ultimately, understanding how FN3K’s substrate preferences are wired into its structure opens new avenues for therapeutic intervention and illuminates the broader principles of kinase substrate specificity.

## Methods

### Plasmids

HsFN3K WT, D217A, W219A, H288A, and H291 were cloned previously^18^. pGro7 was obtained from Takara Bio (#3340). A gene fragment of HsKT3K (Integrated DNA Technologies, listed in Supplementary Table 1) was cloned into the multiple cloning site driven by the polyhedrin promoter of the pFastBac™ Dual vector (Gibco #10712024). The HsKT3K fragment and pFastBac™ Dual vector were digested using BamHI and HindIII and ligated together using T4 DNA Ligase (NEB #M0202S) before transformation into NEB® Turbo cells (#C2984H).

Gene fragments of ancestral proteins and HsFN3K 5^mut^ were codon-optimized for *E. coli* expression (Integrated DNA Technologies, listed in Supplementary Table 1) and cloned into a modified pET15b vector containing a N-terminal maltose-binding protein and His-tag using Gibson assembly. The modified pET15b vector was digested using NdeI and HindIII. The digested vector and codon-optimized gene fragment were combined in a 5:1 molar ratio in a 10 µL reaction in Gibson Assembly® Master Mix (NEB #E2611S) and incubated at 50 °C for 1 h before being transformed into NEB® Turbo cells.

Mutations were introduced into HsFN3K, ancFN3K, and ancFN3KRP through linearization of the WT vector using the primers listed in Supplementary Table 2. Linearized vectors were religated using Gibson assembly before being transformed into NEB® Turbo cells.

### Protein expression and purification

HsFN3K and ancestral proteins were co-expressed with the pGro7 plasmid containing GroEL/GroES in BL21(DE3) *E. coli* cells. Cells were grown to an OD of 0.5–0.7 and induced with 0.5 mM IPTG and 0.5 mg/mL L-arabinose for 16 h at 18 °C. Cells were harvested at 6000 × *g*, lysed, and clarified at 40,000 × *g* in lysis buffer containing 20 mM Tris, pH 8.0, 500 mM NaCl, 20 mM imidazole, 1 mM PMSF, 1 mM DTT. The clarified lysate was then subjected to Ni-NTA (Cytiva #17525501) affinity purification and eluted with a linear gradient of imidazole (20–500 mM imidazole in 50 mM Tris, pH 8.0, 300 mM NaCl, 5 mM BME). Subsequently, MBP-tagged proteins were purified using MBPTrap HP (Cytiva #28918779) and eluted using 50 mM Tris, pH 8.0, 300 mM NaCl, 1% maltose, and 5 mM BME. Unless otherwise stated, MBP-tags were cleaved using rTEV protease. The fusion tag was removed through Ni-NTA purification, and the cleaved protein was subjected to SD200 size-exclusion chromatography in a final buffer of 50 mM HEPES, pH 7.0, 250 mM NaCl, and 1 mM DTT.

KT3K-His was expressed in Sf9 insect cells. Cells were harvested at a viability between 30 and 50%. Cell pellets were washed with ice-cold PBS supplemented with protease inhibitors (Thermo Scientific #A32961) to remove EDTA. Protein was extracted through homogenization in lysis buffer and subsequent centrifugation. Soluble fractions were purified on an AKTA using Ni-NTA affinity chromatography and SD200 size-exclusion chromatography. All proteins were concentrated to ~4 mg/mL and flash frozen in liquid nitrogen. Proteins were stored at −80 °C.

### Lysozyme glycation and colorimetric assay

60 mg/mL lysozyme (Hampton Research #HR7-110) was incubated with 4 M ribose or glucose in deionized water at 50 °C for 72 h or 28 days, respectively. Glycation reactions were quenched through overnight dialysis against water to remove excess free sugars. Due to increased heterogeneity over time, glycated lysozyme was used within 1 week of dialysis. Glycation was evaluated through a nitro-blue tetrazolium (NBT) assay, as previously described^33,34^. Briefly, 50 µM of substrate was incubated with 0.25 mg/ml NBT (Fisher Scientific #N6495) for 30 min at 37 °C. The incubated mixture was diluted with 0.1 M Carbonate/Bicarbonate pH 10.3 (Thomas Scientific #C988L92) to a final concentration of 5 µM of substrate and incubated for an additional 30 min at 37 °C. Reactions were measured at 530 nm using a BioTek Synergy H1 microplate reader (Agilent Technologies). All experiments were performed in triplicate, and the data were plotted using GraphPad Prism.

### Phosphorylated protein staining

The phosphorylation assays were performed as previously stated^18^. Briefly, the kinase reaction was carried out for 1 h at 25 °C in a 10 µL final volume containing 5 µM of HsFN3K or HsKT3K, 200 µM of fructolysozyme, and 1× kinase buffer (5 mM HEPES, pH 7.0, 0.1 µM BSA, 5 mM MgCl_2_, and 100 µM ATP). The reaction was analyzed by SDS-PAGE. The gel was stained with Pro-Q Diamond Phosphoprotein stain and imaged using the BioRad ChemiDoc Pro-Q channel (ex.602nm). The gels were then stained with Coomassie Blue to confirm loading.

### Luciferase-based kinase assay

The kinase reaction was carried out at room temperature in 10 µL containing 200 µM lysozyme (lysozyme, ribulolysozyme, or fructolysozyme) or 100 µM DMF, 100 µM ATP, 5 mM MgCl_2_, 0.1 µM BSA, and 0–5 µM of serially diluted kinase. The reaction was quenched after 30 min using the Kinase-Glo® Plus reagent (Promega #V3771). The final reaction was incubated for 10 min at room temperature. Luminescence was measured using a BioTek Synergy H1 microplate reader (Agilent Technologies). All experiments were performed in triplicate, and the data were plotted using GraphPad Prism. Due to the possible background activity, unmodified lysozyme was used as a background reading and removed.

### Differential scanning fluorimetry

Differential scanning fluorimetry was performed as described previously^18^. Briefly, protein was diluted in 50 mM HEPES, pH 7.0, 250 mM NaCl, 1 mM DTT, and 2 mM MgCl_2_ to a final concentration of 5 µM. ATP was added to a final concentration of 100 µM. 5× SYPRO dye was added before being analyzed using a StepOnePlus Real-Time PCR machine (Applied Biosystems). The mixture was heated from 20 °C to 95 °C at a rate of 1 °C per minute. Melting curves were generated from the first derivative of the fluorescent readings. DSF scans of all samples were performed in triplicate, and the data were plotted using GraphPad Prism.

### Ancestral protein reconstruction

Protein sequences for FN3Ks and KT3Ks were retrieved from UniProt^35^. 42 homologs were identified using the HMMER web server^36^. The homolog sequences were aligned using the PHYLIP interleaved output parameters of MUltiple Sequence Comparison by Log- Expectation (MUSCLE)^37,38^. The resulting multiple sequence alignment was used as input for PhyML 3.0. A phylogeny tree was generated using maximum likelihood estimation, PhyML 3.0, using the substitution model Q.plant +G + I as selected by smart model selection (SMS) using Akaike Information Criterion (AIC)^39,40^. 500 Bootstrap replications were performed. Ancestral sequences were predicted using Phylogenetic Analysis by Maximum Likelihood (PAML) 4.10.1 using the default settings in the CodeML package^41^. The resulting phylogeny tree was visualized using FigTree v.1.4.4 to evaluate nodes of interest. The most probable characters at each site, for each node, were exported for further consideration. Of this reconstruction, four ancestral proteins were selected for further synthesis: ancFN3KRP, ancKT3K-1, ancKT3K-2, and ancFN3K, corresponding to the nodes 46, 47, 48, and 61 using the labeling system in PAML.

### Percent identity matrix

Protein sequences of FN3Ks (94) and KT3Ks (58) were aligned using Clustal Omega^42^. Logo plots were generated using this sequence alignment in WebLogo 3.0^43^. The percent identity output was graphed using GraphPad Prism.

### Protein structure and network predictions

The structures of ancFN3K and HsKT3K were predicted using AlphaFold3^44^. Protein Structure Network analyses were done using webPSN^45^. Intramolecular forces were assessed using ProteinTools^46^.

### Statistics and reproducibility

For the gain-of-function and loss-of-function mutations, a one-way ANOVA was performed with a Dunnett’s multiple comparisons test. The alpha level was set to 0.05. Each data point represents the means of technical triplicates, and error bars indicate standard error. *P*-value was determined using Dunnett’s multiple comparisons test.

## Supplementary information

Supplementary Information

Description of Additional Supplementary Materials

Supplementary Data

## Data Availability

All data supporting the findings of this study are available within the paper and its Supplementary Information. Ancestral reconstruction and plasmid sequence data are available through Zenodo (10.5281/zenodo.17965681). Uncropped blot images can be found in Supplementary Fig. 11. Experimental data generated in this study is available in Supplementary Data. Previously published structures used in this study can be accessed using PDB codes 8UE1 and 9CX8.
