# Supplementary material for: Ancestral protein reconstruction reveals the mechanism of substrate specificity in FN3K-mediated deglycation
Source: Commun Biol. 2026 Apr 3;9:738. doi: 10.1038/s42003-026-09967-3 (PMC13223202; doi:10.1038/s42003-026-09967-3)
Supplement: Supplementary file 1 — Supplementary Information [file 42003_2026_9967_MOESM1_ESM.pdf]

Supplementary Information

Ancestral protein reconstruction reveals the mechanism of substrate specificity in FN3K-mediated deglycation.

Jenet K. Matlack[1,2], Robert E. Miner III[2,3], Jameela Lokhandwala[2], Jennifer M. Binning[2]

[1]Department of Molecular Medicine, Morsani College of Medicine, University of South Florida, Tampa, Florida, 33612, USA.

[2]Department of Molecular Oncology, H. Lee Moffitt Cancer Center and Research Institute, Tampa, Florida 33612, USA.

[3]Cancer Biology PhD Program, University of South Florida, Tampa, Florida, 33612, USA.

*Correspondence should be addressed to Jennifer M. Binning, (813)745-4778, (jennifer.binning@moffitt.org)

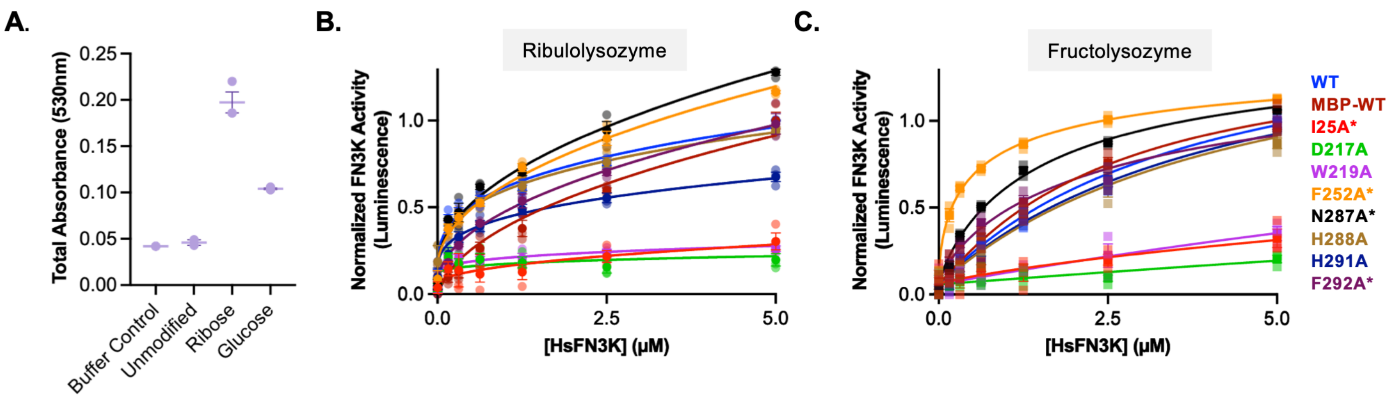

D.

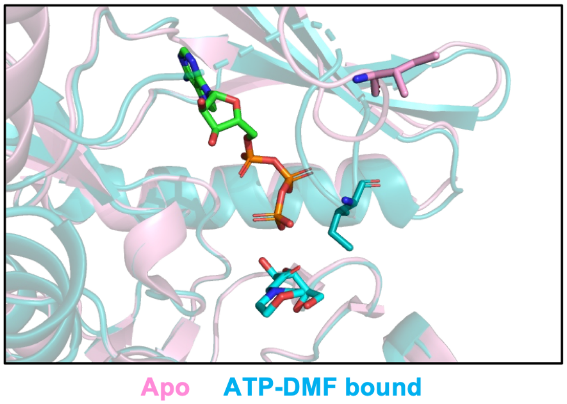

E.

Protein	+ATP (Δ˚C)
FN3K	+4.2
I25A	+0.0
D217A	+2.3
W219A	+5.1
F252A	+2.3
N287A	+0.8
H288A	+4.2
H291A	+3.1
F292A	+1.5

Supplemental Figure 1. Kinase activity of substrate binding mutations in HsFN3K. **A.** NBT colorimetric assay of buffer control, unmodified lysozyme, ribose-glycated lysozyme, and glucose-glycated lysozyme. **B-C.** Kinase assay of HsFN3K mutants on fructolysozyme (B) and ribulolysozyme (C). Each data point represents means of technical triplicates; error bars indicate standard error. Asterisks (*) represent protein retaining a MBP-tag. **D.** HsFN3K I25 position in apo (PDB: 9CX8, pink) and ATP-DMF bound (PDB: 9CXM, blue). **E.** Change in melting temperatures of FN3K mutants in the presence of ATP. Each value represents means of technical triplicates.

A.

Residue	Conservation (%)
I25	91
G152	97
D217	100
W219	97
F252	87
N287	100
H288	95
H291	86
F292	98

B.

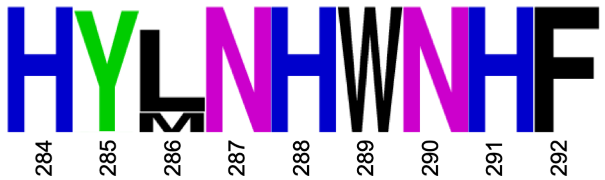

C.

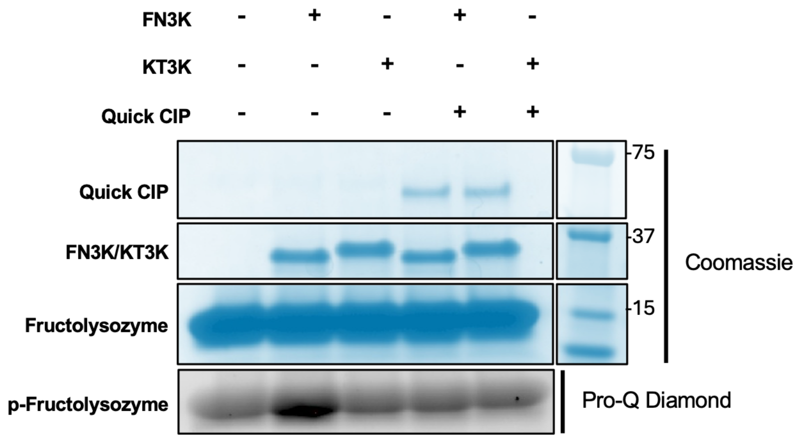

Supplemental Figure 2. FN3K family conservation. A. Percent conservation of each residue identified in protein-substrate interaction analysis. Conservation was determined by ConSurf. **B.** Logo plot of substrate binding residues in KT3Ks. **C.** SDS-PAGE analysis of fructolysozyme treated with HsFN3K and HsKT3K. The gels were stained with a phosphostain to monitor phosphorylation and Coomassie blue to monitor total protein.

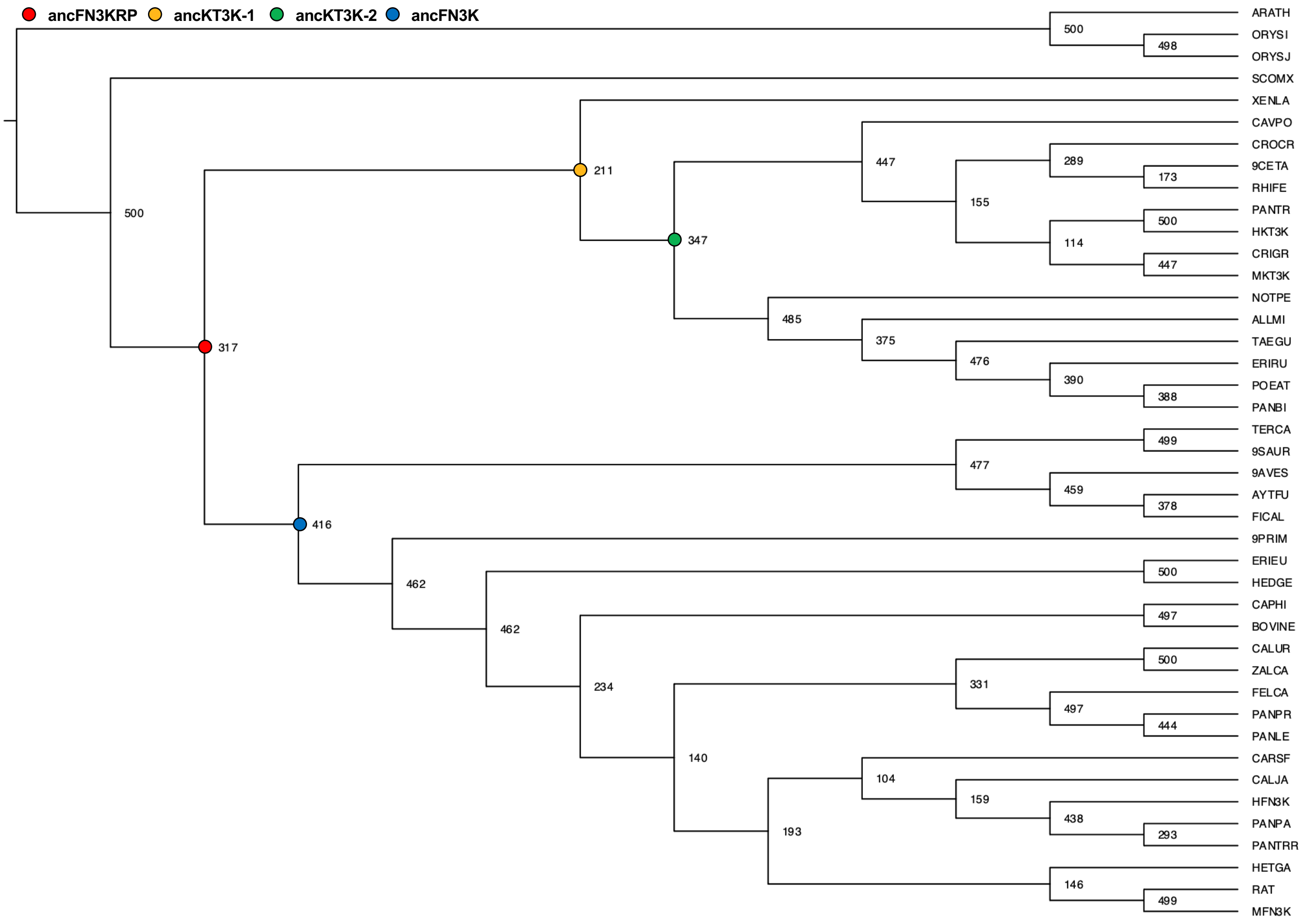

Supplemental Figure 3. Phylogenetic analysis of FN3K family for ancestral protein reconstruction. Bootstrap values (B=500) are indicated at each node. Ancestral proteins of interest are labeled at their respective nodes. B: Bootstrap replications.

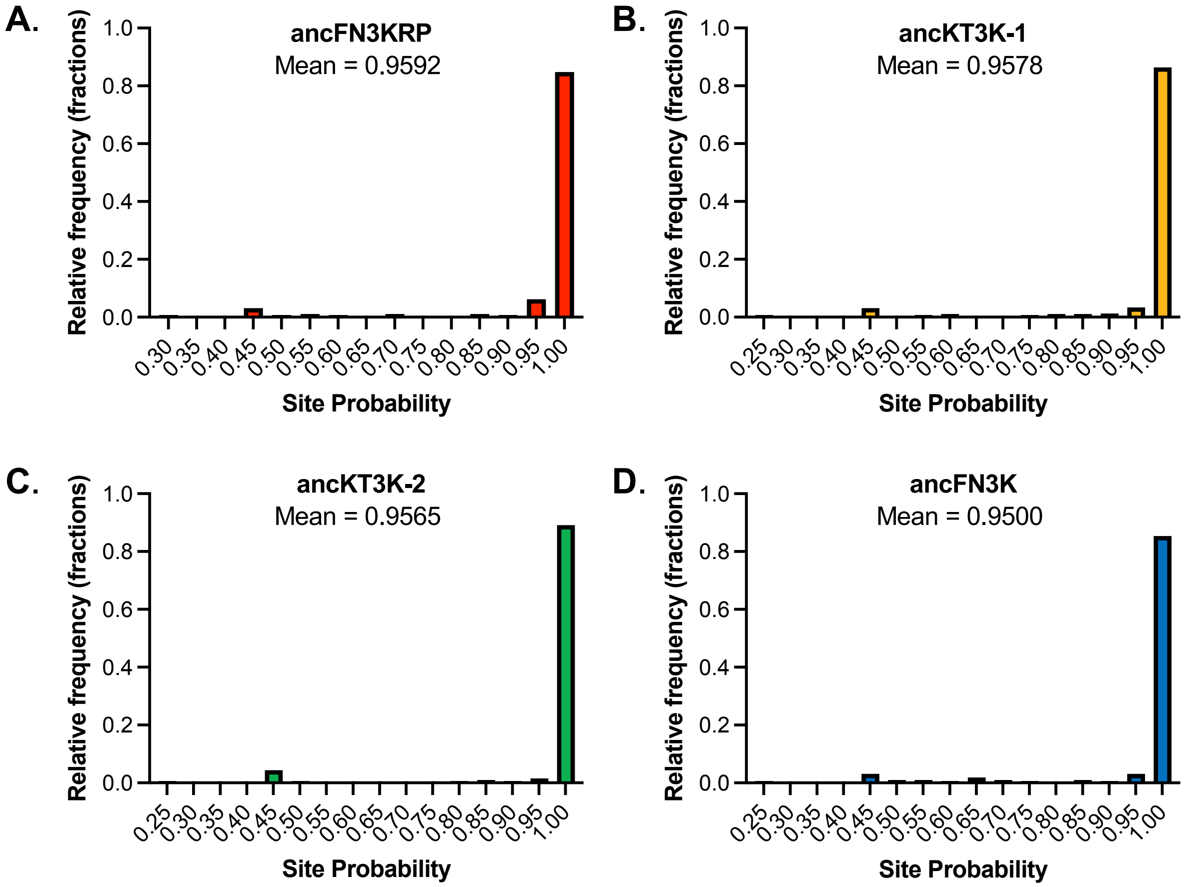

Supplemental Figure 4. Histogram of posterior probabilities from PAML reconstruction for most common amino acid at each site. A-D. Mean probability across all sites in listed below each protein of interest.

A.

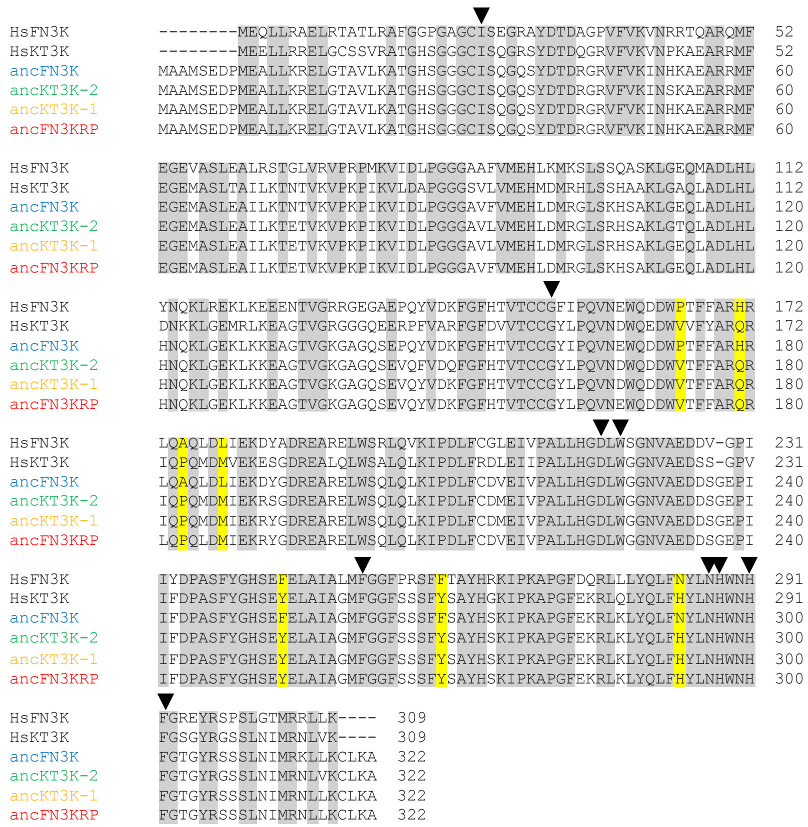

B.

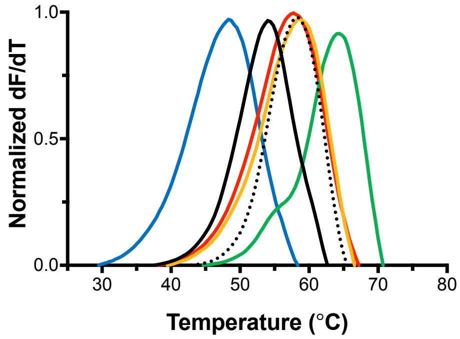

C.

HsFN3K	54.4 ± 0.010˚C
HsKT3K	58.4 ± 0.005˚C
ancFN3KRP	57.7 ± 0.011˚C
ancKT3K-1	58.7 ± 0.004˚C
ancKT3K-2	64.8 ± 0.015˚C
ancFN3K	47.9 ± 0.003˚C

Supplemental Figure 5. Ancestral reconstruction of fructosamine kinase family generates stable proteins with high homogeneity. A. Multiple sequence alignment of HsFN3K, HsKT3K, ancFN3KRP, ancKT3K, ancKT3K, and ancFN3K. Conserved amino acid residues across all proteins are highlighted in gray. Amino acid residues found only in KT3K-like proteins are highlighted in yellow. Substrate binding residues from Figure 1 are indicated by black arrows. **B.** Normalized DSF melt curves of HsFN3K (black solid), HsKT3K (dotted), ancFN3KRP (red), ancKT3K-1 (yellow), ancKT3K-2 (green), and ancFN3K (blue). Curves represent the means of technical triplicates. **C.** Melting temperatures determined in (B) with standard deviation.

A.

Site	ancFN3KRP	ancFN3K	KT3K	FN3K	Site
74	E	N	N	G	66
145	V	P	R	P	137
162	L	I	L	I	154
173	V	P	V	P	165
179	Q	H	Q	H	171
183	P	A	P	A	175
187	M	L	M	L	179
191	R	D	E	D	183
253	Y	F	Y	F	244
269	Y	F	Y	F	260
293	H	N	H	N	284
315	N	K	N	R	306

B.

Site	ancFN3KRP	ancKT3K-1	ancKT3K-2	ancFN3K
74	E (0.722)	E (0.735)	Q (0.377)	N (0.942)
145	V (0.954)	V (0.959)	V (0.970)	P (0.967)
162	L (0.575)	L (0.579)	L (0.996)	I (0.991)
173	V (0.993)	V (1.000)	V (1.000)	P (0.985)
179	Q (0.976)	Q (1.000)	Q (1.000)	H (0.985)
183	P (0.617)	P (0.993)	P (1.000)	A (0.989)
187	M (0.941)	M (0.999)	M (1.000)	L (0.986)
191	D (0.491)	D (0.329)	N (0.452)	D (0.977)
253	Y (0.575)	Y (0.579)	Y (0.996)	F (0.991)
269	Y (0.997)	Y (1.000)	Y (1.000)	F (0.985)
293	H (0.997)	H (1.000)	H (1.000)	N (0.985)
315	N (0.981)	N (0.987)	N (1.000)	K (0.957)

C.

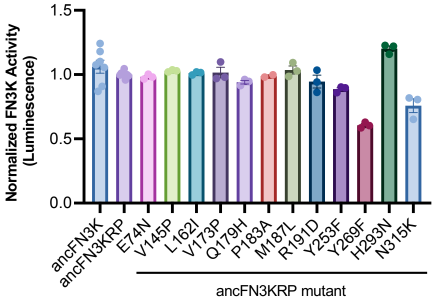

D.

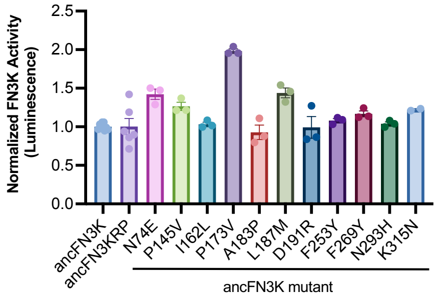

Supplemental Figure 6. Switch mutations in ancFN3Ks. A. The 12 amino acid residues that differ between ancFN3KRP and ancFN3K and their corresponding residues in HsFN3K and HsKT3K. **B.** Posterior probabilities of the 12 residues that differ between ancFN3KRP and ancFN3K across the 4 ancestral proteins. **C-D.** Kinase activity of ancFN3KRP mutants (C) and ancFN3K mutants (D) towards ribulolysozyme normalized to their respective WT enzyme. Each bar graph represents the activity at 5 μM of enzyme. Each bar represents means of technical triplicates except for: ancFN3KRP WT where (C) n=8 and (D) n=6; ancFN3K WT where (C) n=7 and (D) n=9; error bars indicate standard error.

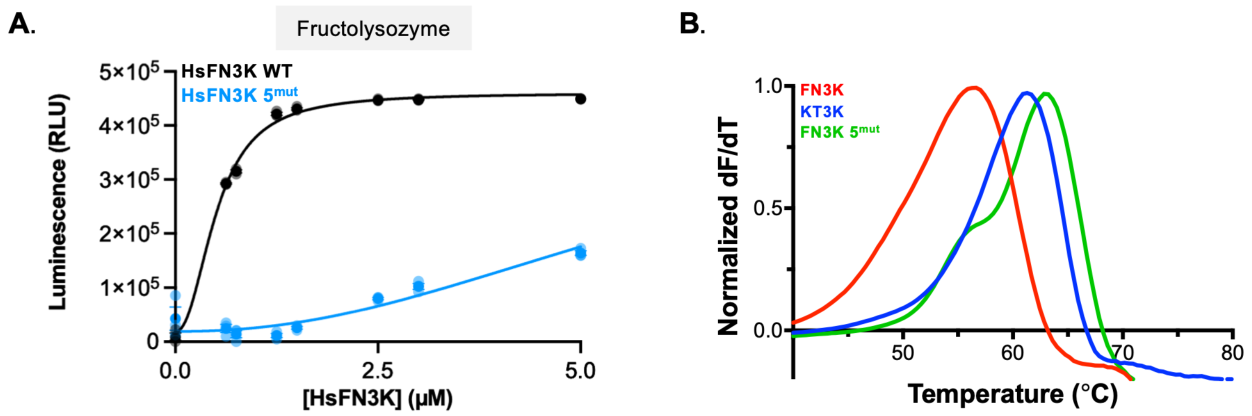

Supplemental Figure 7. Mutations in FN3K make them more KT3K-like. A. Kinase assay of HsFN3K WT and 5mut towards fructolysozyme. Each data point represents means of technical triplicates; error bars indicate standard error. **B.** DSF melt curve of HsFN3K, HsKT3K, and HsFN3K 5^{mut}. Curves represent the means of technical triplicates.

A.

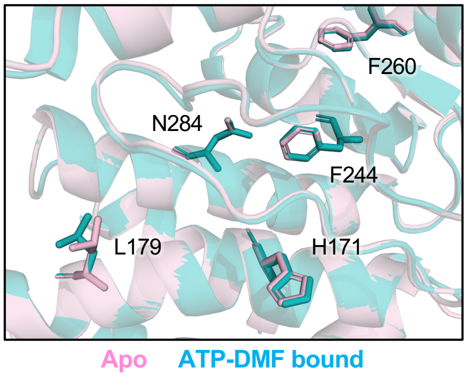

B.

HsFN3K N284	
F283	1.33
Y285	1.34
**H288**	**2.91**
Q281	2.97
Y280	3.08
L282	3.19
**N287**	**3.30**
L286	3.33
M251	3.49
I248	3.64
A247	4.06
**F244**	**4.29**
**F252**	**4.79**
W289	4.99
**D217**	**5.16**
L279	5.73
Y296	5.86

HsKT3K H284	
F283	1.33
Y285	1.34
**H288**	**2.83**
Q281	3.13
Y280	3.30
L282	3.31
**Y244**	**3.32**
L286	3.41
**N287**	**3.43**
I248	3.53
M251	3.79
G216	4.03
A247	4.14
C151	4.71
**D217**	**4.75**
W289	5.01
**F252**	**5.20**
Y168	5.82
Y296	5.93
E245	5.93

Supplemental Figure 8. Intramolecular contact analysis of HsFN3K and HsKT3K. A. HsFN3K apo (pink, PDB: 9CX8) versus ATP-DMF bound (blue, PDB: 9CXM) structures. H171, L179, F244, F260, and N284 are represented as sticks. **B.** Intramolecular contact analysis of HsFN3K N284 (B) and AlphaFold predicted HsKT3K H284 (C). Purple represents established substrate-binding residues. Pink represents residues highlighted in this study.

A. AtFN3K HsFN3K Shared

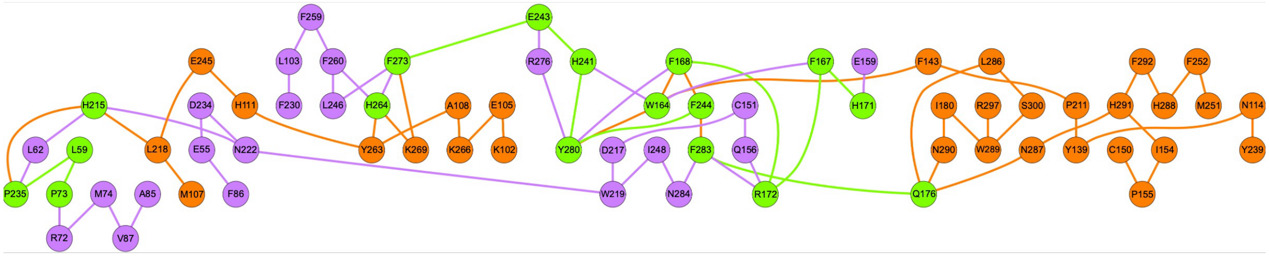

B.

```
AtFN3K      MAVASLSICFSARPHLLLRNFSPRPKFVAMAAMSEDPIREWILTEGKATQITKIGSVGGG    60
HsFN3K      -------------------------------------MEQLLRAELRTATLRAFGGPGAG    23
                                                 :.: : :* ::: :  :*. *.*

AtFN3K      CINLASHYQTDAGSFFVKTNRS-IGPAMFEGEALGLEAMYETRTIRVPNPHKAGELPTGG    119
HsFN3K      CISEGRAYDTDAGPVFVKVNRRTQARQMFEGEVASLEALRSTGLVRVPRPMKVIDLPGGG    83
            **. .  *:**** .***.**   .  *****. .***: .*  :***.* *. :** **

AtFN3K      SYIIMEFIDFGGSRGNQAELGRKLAEMHKAG-----------------------KTSKGF    156
HsFN3K      AAFVMEHLKMKSLSSQASKLGEQMADLHLYNQKLREKLKEEENTVGRRGEGAEPQYVDKF    143
            : ::**.:.: .  .: ::**.::*::*  .                       :  . *

AtFN3K      GFEVDNTIGSTPQINTWSSDWIEFYGEKRLGYQLKLARDQYGDSAIYQKGHTLIQNMAPL    216
HsFN3K      GFHTVTCCGFIPQVNEWQDDWPTFFARHRLQAQLDLIEKDYADREARELWSRLQVKIPDL    203
            **.. .  *  **:* *..** *:..:** **.* ..:*.*    :    *  ::  *

AtFN3K      FENVVIEPCLLHGDLWSGNIAYDKNNEPVILDPACYYGHNEADFGMSW-CAGFGESFYNA    275
HsFN3K      FCGLEIVPALLHGDLWSGNVAED-DVGPIIYDPASFYGHSEFELAIALMFGGFPRSFFTA    262
            * .: * *.*********:* * :  *:* ***.:***.* ::.::   .** .**:.*

AtFN3K      YFKVMPKQAGYEKRRDLYLLYHYLNHYNLFGSGYRSSAMSIIDDYLRMLKA      326
HsFN3K      YHRKIPKAPGFDQRLLLYQLFNYLNHWNHFGREYRSPSLGTMRRLLK----      309
            *.: :**  *::*  ** *::****:* **  *** ::. :   *:
```

Supplemental Figure 9. Protein Structure Network analysis of HsFN3K and AtFN3K. A. Difference PSN of AtFN3K (PDB: 6OID, purple) and HsFN3K (PDB: 9CX8, orange). Shared residues in each network are green. Residues are labeled as HsFN3K residues. **B.** Multiple sequence alignment of HsFN3K and AtFN3K. Yellow box highlights alpha-helix where F244 resides.

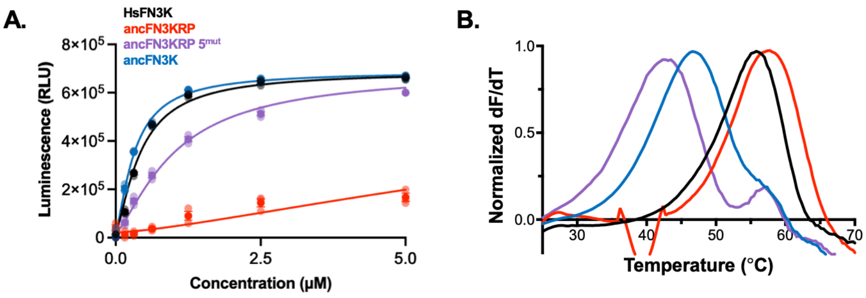

C.

HsFN3K	56.2 ± 0.010˚C
ancFN3KRP	57.7 ± 0.007˚C
ancFN3KRP 5^{mut}	41.5 ± 0.015˚C
ancFN3K	46.2 ± 0.003˚C

Supplemental Figure 10. Neofunctionalization of fructosamine repair by FN3Ks begins with a destabilization event. A. Kinase assay of ancFN3KRP, ancFN3KRP 5^{mut}, ancFN3K, and HsFN3K against DMF. Each data point represents means of technical triplicates; error bars indicate standard error. **B**. DSF melting curves and corresponding melting temperatures for ancFN3KRP, ancFN3KRP 5^{mut}, ancFN3K, and HsFN3K. Curves represent the means of technical triplicates. **C.** Melting temperatures determined in (B) with standard deviation.

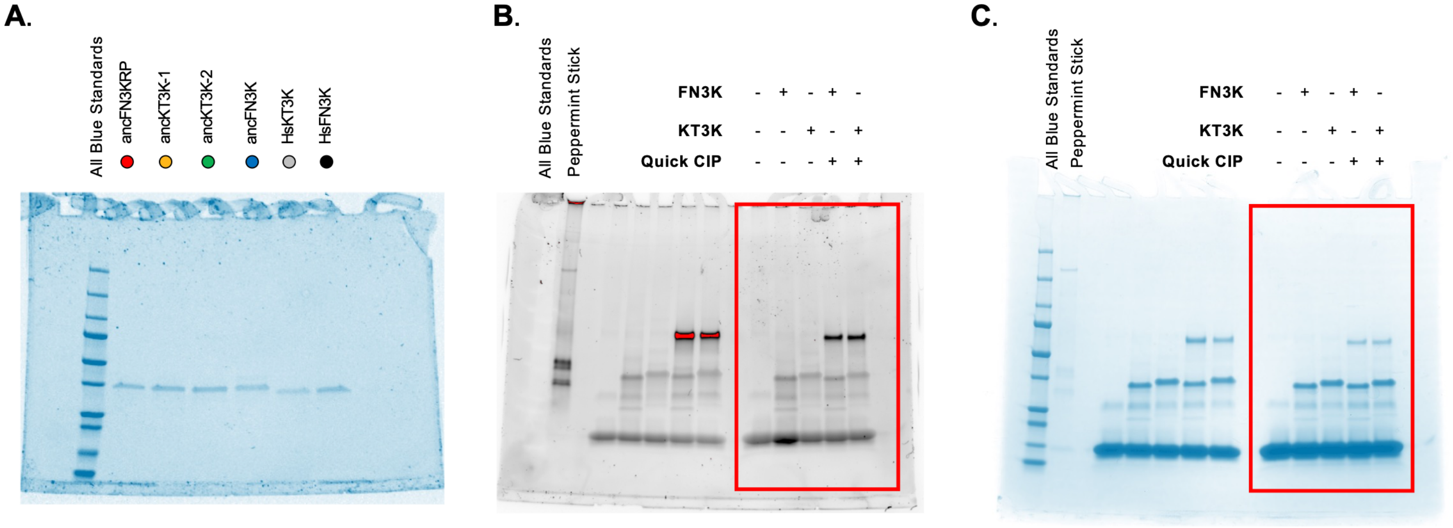

Supplemental Figure 11. Raw SDS-PAGEs of Figures 3B and S2C. A. Coomassie stain of ancestral and extant FN3Ks and KT3Ks. Figure highlighted in red. **B.** Pro-Q Diamond stain of lysozyme phosphorylation. **C.** Coomassie stain of total protein. Figure highlighted in red.

**Gene Fragments**
**>HsKT3K**<br>ATGGGCAGCAGCCATCATCATCATCATCACAGCAGCGGCCTGGTGCCGCGCGG<br>CAGCCATATAGAAAACCTGTATTTTCAGGGCCATATGGAAGAGTTATTACGTCGC<br>GAGCTTGGCTGCAGTAGTGTGCGTGCTACTGGTCATTCTGGCGGGGGATGCATT<br>AGTCAAGGCCGCTCTTATGACACCGATCAGGGTCGCGTCTTTGTCAAGGTCAAT<br>CCCAAGGCAGAAGCGCGTCGCATGTTTGAGGGAGAGATGGCTTCTCTGACAGC<br>AATCTTGAAAACTAATACGGTCAAGGTGCCAAAACCGATCAAAGTCCTGGACGC<br>CCCAGGTGGGGGTTCCGTGCTGGTAATGGAACACATGGATATGCGCCACTTGTC<br>CTCACATGCTGCGAAATTAGGAGCCCAGCTGGCGGATTTACATCTTGATAATAAA<br>AAGTTAGGAGAAATGCGTCTGAAAGAGGCAGGTACAGTAGGACGTGGAGGAGG<br>ACAAGAAGAACGTCCCTTCGTAGCGCGTTTCGGGTTCGACGTAGTCACCTGTTG<br>CGGTTATTTGCCGCAGGTGAACGATTGGCAAGAAGATTGGGTAGTCTTTTACGC<br>CCGCCAGCGTATCCAGCCTCAAATGGACATGGTAGAAAAAGAGTCTGGTGATCG<br>CGAGGCCTTACAACTGTGGTCCGCGCTTCAGCTGAAAATCCCTGATTTATTCCGT<br>GACTTAGAAATTATTCCCGCACTGCTGCATGGTGATCTTTGGGGAGGAAACGTT<br>GCTGAAGATAGTAGCGGCCCTGTCATCTTCGATCCAGCGTCTTTTTATGGGCACT<br>CCGAGTACGAATTAGCTATCGCCGGAATGTTCGGAGGTTTTTCCTCTTCATTTTA<br>TAGCGCGTATCACGGGAAGATTCCCAAAGCGCCAGGTTTCGAGAAGCGTCTTCA<br>GTTATACCAGCTTTTCCATTATCTGAACCATTGGAATCATTTTGGGTCTGGGTACC<br>GTGGGTCGTCATTAAATATCATGCGCAATCTTGTGAAGTAGGGATCCGGCTGCT<br>AACAAAGCCCGAAAGGAAGCTGAGTTGGCTGCTGCCACCGCTGAGCAATAACTA<br>GCATAACCCCTTGGGGCCTCTAAACGGGTCTTGAGGGGTTTTTTG
**>ancFN3KRP**<br>CATATAGAAAACCTGTATTTTCAGGGCCATATGGCTGCTATGAGCGAAGATCCTA<br>TGGAGGCGCTTCTGAAACGTGAGCTTGGTACCGCGGTTTTAAAGGCGACGGGA<br>CATTCTGGTGGCGGGTGCATTAGCCAGGGGCAAAGTTATGACACGGACCGTGG<br>TCGTGTTTTTGTAAAGATTAACCATAAGGCAGAAGCGCGTCGTATGTTCGAGGGT<br>GAGATGGCATCCCTTGAGGCGATCCTTAAAACCGAAACTGTCAAAGTTCCGAAA<br>CCGATCAAAGTGATCGACTTACCTGGGGGTGGCGCCGTCTTCGTAATGGAGCAT<br>TTGGATATGCGCGGACTTTCAAAACATTCGGCTAAGTTGGGAGAACAATTAGCG<br>GATCTTCACCTGCACAACCAAAAGTTGGGTGAAAAATTGAAGAAGGAAGCAGGC<br>ACGGTAGGCAAAGGAGCGGGACAGAGTGAAGTCCAGTATGTAGATAAGTTCGG<br>GTTTCACACGGTTACGTGTTGCGGGTACCTTCCACAGGTCAATGAGTGGCAGGA<br>CGACTGGGTGACTTTTTTCGCACGTCAGCGCTTACAACCCCAACTGGACATGAT<br>CGAGAAACGCTATGGGGACCGCGAGGCCCGCGAGCTGTGGAGCCAGCTTCAAT<br>TAAAGATCCCTGACTTGTTCTGTGATGTCGAAATCGTACCCGCACTTCTTCATGG<br>CGATTTGTGGGGAGGTAATGTGGCCGAGGATGACTCGGGTGAACCGATCATTTT<br>CGACCCCGCAAGTTTCTACGGCCATTCCGAGTACGAGCTGGCTATCGCGGGAAT<br>GTTTGGCGGATTCTCCTCGTCCTTCTACTCAGCTTACCACAGCAAAATTCCGAAA<br>GCACCAGGCTTTGAGAAGCGTTTAAAATTGTACCAATTATTTCACTACCTTAACCA<br>TTGGAATCATTTCGGCACGGGATACCGCTCCAGTAGTCTTAATATTATGCGTAAC<br>TTGTTGAAGTGCTTAAAAGCCTAGAAGCTTTCTAGACTCGAGCCCGGGGGTCCG
**>ancKT3K-1**<br>CATATAGAAAACCTGTATTTTCAGGGCCATATGGCTGCCATGAGCGAAGATCCAA

TGGAAGCATTATTGAAACGCGAATTGGGCACAGCCGTGCTTAAGGCTACCGGTC<br>ATAGCGGCGGTGGATGCATCAGTCAGGGACAGAGTTACGATACTGATCGTGGC<br>CGCGTGTTTGTCAAAATCAATCACAAGGCCGAAGCCCGCCGTATGTTCGAAGGT<br>GAGATGGCCAGTTTAGAAGCGATTCTGAAAACGGAGACTGTAAAGGTACCTAAA<br>CCGATTAAGGTGATCGACTTACCTGGCGGAGGAGCCGTGTTAGTAATGGAGCAC<br>CTTGACATGCGCGGTCTGTCTCGCCATTCCGCTAAGTTAGGCGAACAGTTAGCA<br>GACTTACATTTACATAATCAGAAATTAGGAGAGAAATTAAAAAAAGAAGCGGGTA<br>CTGTAGGCAAAGGAGCCGGACAGTCTGAAGTTCAATATGTAGACAAATTCGGTTT<br>CCATACTGTCACCTGTTGCGGGTACTTGCCGCAAGTCAACGACTGGCAAGATGA<br>CTGGGTAACATTCTTCGCCCGTCAGCGCATCCAGCCCCAAATGGACATGATCGA<br>GAAACGTTATGGCGACCGTGAAGCCCGCGAGCTTTGGTCGCAGTTACAACTGAA<br>AATCCCAGACTTATTTTGTGATATGGAGATCGTTCCCGCGCTGTTACATGGAGAC<br>TTATGGGGTGGTAATGTCGCGGAGGATGACAGTGGGGAGCCCATCATTTTTGAT<br>CCGGCATCATTCTATGGTCATTCAGAGTATGAGTTGGCCATTGCCGGGATGTTC<br>GGCGGATTCTCGTCGAGTTTTTATTCTGCATATCATTCGAAGATCCCGAAAGCGC<br>CTGGATTTGAAAAACGCTTGAAGTTATACCAGTTATTCCACTACTTGAACCACTG<br>GAATCATTTCGGTACAGGATATCGCAGTTCGTCCCTTAACATTATGCGTAATCTT<br>CTGAAATGTTTGAAGGCCTAGAAGCTTTCTAGACTCGAGCCCGGGGGTCCG
**>ancKT3K-2**<br>CATATAGAAAACCTGTATTTTCAGGGCCATATGGCAGCTATGAGCGAAGACCCAA<br>TGGAGGCGTTGCTGAAACGTGAATTGGGTACAGCCGTTCTTAAAGCCACAGGGC<br>ATAGTGGCGGAGGATGTATCAGCCAAGGCCAAAGCTATGATACGGATCGCGGTC<br>GCGTGTTCGTGAAGATTAACAGTAAAGCGGAAGCCCGCCGTATGTTTGAAGGGG<br>AAATGGCTTCGCTTGAGGCCATCCTTAAGACGGAGACAGTGAAAGTACCTAAGC<br>CGATTAAGGTCATTGATTTGCCAGGCGGTGGGGCGGTGCTTGTGATGGAACACT<br>TGGACATGCGTGGGCTGTCACGTCATAGTGCCAAATTAGGAACGCAGCTGGCAG<br>ACTTACATCTTCACAACCAAAAATTAGGGGAAAAATTGAAAAAGGAGGCCGGAAC<br>TGTTGGAAAGGGTGCTGGGCAGTCAGAAGTTCAGTTCGTGGACCAGTTCGGGTT<br>CCACACCGTGACCTGTTGCGGATATTTGCCGCAGGTGAACGATTGGCAAGACGA<br>TTGGGTGACGTTCTTCGCACGTCAGCGCATCCAGCCACAGATGGATATGATCGA<br>GAAACGCAGTGGAGACCGTGAAGCGCGCGAATTATGGAGCCAGCTTCAGCTTA<br>AGATTCCCGATTTATTTTGCGACATGGAGATTGTACCCGCTCTGTTGCATGGCGA<br>CTTGTGGGGTGGGAATGTCGCCGAAGATGATTCCGGGGAGCCCATTATTTTTGA<br>CCCGGCGTCCTTTTACGGGCACAGCGAATATGAATTAGCCATTGCCGGTATGTT<br>CGGTGGTTTCTCCTCCTCCTTTTATTCCGCTTATCACAGTAAGATCCCCAAAGCG<br>CCAGGTTTTGAGAAGCGTTTAAAACTGTACCAACTTTTCCATTATTTGAATCACTG<br>GAACCATTTCGGCACTGGCTATCGTGGCTCTTCATTAAATATCATGCGTAACTTA<br>GTAAAATGCTTAAAGGCATAGAAGCTTTCTAGACTCGAGCCCGGGGGTCCG
**>ancFN3K**<br>CATATAGAAAACCTGTATTTTCAGGGCCATATGGCAGCGATGTCAGAAGACCCCA<br>TGGAGGCTCTGCTTAAACGCGAGTTAGGAACAGCTGTACTGAAGGCTACGGGCC<br>ACAGTGGAGGTGGTTGTATTTCCCAGGGGCAGAGCTACGACACTGACCGTGGG<br>CGTGTATTCGTAAAGATCAATCATAAGGCCGAAGCCCGTCGCATGTTTGAGGGC<br>GAAATGGCCTCGCTGGAGGCCATTCTGAAAACAAATACCGTAAAAGTACCGAAG<br>CCTATCAAGGTAATCGACTTGCCAGGAGGAGGGGCCGTATTTGTGATGGAGCAT<br>TTGGATATGCGTGGATTGAGTAAGCACTCCGCCAAACTTGGCGAGCAACTTGCT

GATTTACATTTACACAATCAGAAGCTGGGGGAGAAATTGAAGAAAGAAGCCGGA
ACAGTCGGCAAGGGTGCTGGTCAATCGGAACCCCAATATGTGGATAAATTCGGT
TTTCATACAGTGACTTGCTGTGGATACATTCCTCAAGTGAACGAGTGGCAAGATG
ACTGGCCGACCTTTTTCGCGCGTCACCGCTTGCAAGCGCAGCTTGACTTAATTG
AGAAGGACTATGGTGACCGCGAAGCACGTGAATTATGGAGCCAGCTTCAATTAA
AAATCCCGGATCTTTTCTGCGATGTGGAAATCGTACCAGCCCTGCTTCACGGTG
ACTTGTGGGGTGGGAATGTGGCCGAAGATGACTCAGGTGAACCCATTATCTTTG
ATCCTGCATCCTTCTATGGACACTCGGAGTTTGAATTAGCGATCGCTGGTATGTT
TGGGGGCTTTAGCAGTAGTTTTTTTCTGCATACCATTCAAAGATCCCAAAAGCA
CCAGGATTCGAGAAGCGTTTGAAATTATACCAACTTTTCAATTACCTTAATCATTG
GAACCATTTTGGGACCGGGTATCGTAGTAGTTCGCTTAACATTATGCGTAAATTG
CTTAAATGTTTGAAAGCGTAGAAGCTTTCTAGACTCGAGCCCGGGGGTCCG

>ancFN3KRP 5^{mut}

CATATAGAAAACCTGTATTTTCAGGGCCATATGGCTGCTATGAGCGAAGATCCTA
TGGAGGCGCTTCTGAAACGTGAGCTTGGTACCGCGGTTTTAAAGGCGACGGGA
CATTCTGGTGGCGGGTGCATTAGCCAGGGGCAAAGTTATGACACGGACCGTGG
TCGTGTTTTTGTAAAGATTAACCATAAGGCAGAAGCGCGTCGTATGTTCGAGGGT
GAGATGGCATCCCTTGAGGCGATCCTTAAAACCGAAACTGTCAAAGTTCCGAAA
CCGATCAAAGTGATCGACTTACCTGGGGGTGGCGCNGTCTTCGTAATGGAGCAT
TTGGATATGCGCGGACTTTCAAAACATTCGGCTAAGTTGGGAGAACAATTAGCG
GATCTTCACCTGCACAACCAAAAGTTGGGTGAAAAATTGAAGAAGGAAGCAGGC
ACGGTAGGCAAAGGAGCGGGACAGAGTGAAGTCCAGTATGTAGATAAGTTCGG
GTTTCACACGGTTACGTGTTGCGGGTACCTTCCACAGGTCAATGAGTGGCAGGA
CGACTGGGTGACTTTTTTCGCACGTCACCGCTTACAACCCCAACTGGACCTTATC
GAGAAACGCTATGGGGACCGCGAGGCCCGCGAGCTGTGGAGCCAGCTTCAATT
AAAGATCCCTGACTTGTTCTGTGATGTCGAAATCGTACCCGCACTTCTTCATGGC
GATTTGTGGGGAGGTAATGTGGCCGAGGATGACTCGGGTGAACCGATCATTTTC
GACCCCGCAAGTTTCTACGGCCATTCCGAGTTTGAGCTGGCTATCGCGGGAATG
TTTGGCGGATTCTCCTCGTCCTTCTTCTCAGCTTACCACAGCAAAATTCCGAAAG
CACCAGGCTTTGAGAAGCGTTTAAAATTGTACCAATTATTTAACTACCTTAACCAT
TGGAATCATTTCGGCACGGGATACCGCTCCAGTAGTCTTAATATTATGCGTAACT
TGTTGAAGTGCTTAAAAGCCTAGAAGCTTTCTAGACTCGAGCCCGGGGGTCCG

>HsFN3K 5^{mut}

CATATAGAAAACCTGTATTTTCAGGGCCATATGGAGCAGCTTCTGCGTGCTGAGC
TTCGCACAGCGACTCTGCGCGCATTTGGGGGTCCCGGCGCAGGGTGTATTAGC
GAAGGCCGTGCTTACGACACGGATGCTGGTCCCGTATTCGTTAAGGTTAATCGC
CGCACTCAGGCTCGCCAAATGTTCGAGGGCGAAGTTGCAAGTTTAGAGGCTCTT
CGTAGCACCGGTTTGGTGCGCGTACCCCGCCCTATGAAAGTAATCGACTTGCCC
GGAGGCGGAGCGGCATTTGTGATGGAACACCTTAAAATGAAGTCACTTAGTTCT
CAGGCTTCCAAATTGGGAGAGCAAATGGCCGACTTGCACCTTTACAATCAGAAG
CTGCGCGAAAAGTTAAAAGAAGAGGAGAACACCGTCGGACGCCGCGGCGAGGG
AGCTGAGCCTCAGTATGTAGACAAGTTTGGATTTCACACGGTCACGTGTTGTGG
CTTTATTCCACAGGTGAACGAGTGGCAAGACGACTGGCCTACGTTTTTTGCTCGT
CAACGTCTGCAGGCCCAACTGGATATGATCGAAAAAGATTATGCCGATCGTGAA
GCGCGCGAATTATGGAGCCGCCTTCAAGTCAAAATCCCAGATCTTTTCTGTGGG
CTGGAGATCGTACCTGCATTATTGCATGGCGATCTTTGGTCGGGAAATGTGGCC

GAAGATGACGTAGGGCCTATTATTTATGATCCTGCGAGTTTCTATGGACACTCCG<br>AGTATGAACTTGCTATCGCTTTAATGTTCGGTGGGTTTCCCCGTAGTTTTTACAC<br>GGCGTACCATCGTAAGATCCCTAAAGCCCCAGGTTTCGACCAGCGTCTGCTTCT<br>GTATCAACTTTTTCATTACTTAAACCATTGGAACCATTTCGGTCGTGAATACCGCT<br>CGCCATCGCTGGGTACGATGCGTCGCCTGTTGAAGTAGAAGCTTTCTAGACTCG<br>AGCCCGGGGGTCCG

Supplementary Table 1. A list of gene fragments used in the generation of expression plasmids of ancFN3Ks, HsKT3K, and HsFN3K 5^{mut}.

Primer	Nucleic acid sequence of primers (5'→3')
ancFN3KRP E74N Forward Primer	TAAAACCAACACTGTCAAAGTTCCGAAACCG
ancFN3KRP E74N Reverse Primer	ACAGTGTTGGTTTTAAGGATCGCCTCAAGGG
ancFN3KRP V145P Forward Primer	GAGTGAACCTCAGTATGTAGATAAGTTCGGGTTTC
ancFN3KRP V145P Reverse Primer	TACTGAGGTTCACTCTGTCCCGCTCC
ancFN3KRP L162I Forward Primer	CGGGTACATCCCACAGGTCAATGAGTGGC
ancFN3KRP L162I Reverse Primer	TGTGGGATGTACCCGCAACACGTAACCG
ancFN3KRP V173P Forward Primer	CGACTGGCCAACTTTTTTCGCACGTCAGC
ancFN3KRP V173P Reverse Primer	AAAGTTGGCCAGTCGTCCTGCCACTC
ancFN3KRP Q179H Forward Primer	CGCACGTCACCGCTTACAACCCCAACTGGAC
ancFN3KRP Q179H Reverse Primer	AAGCGGTGACGTGCGAAAAAAGTCACCCAG
ancFN3KRP P183A Forward Primer	CTTACAAGCACAACTGGACATGATCGAGAAACGC
ancFN3KRP P183A Reverse Primer	AGTTGTGCTTGTAAGCGCTGACGTGC
ancFN3KRP M187L Forward Primer	ACTGGACCTTATCGAGAAACGCTATGGGGACC
ancFN3KRP M187L Reverse Primer	TCGATAAGGTCCAGTTGGGGTTGTAAGCG
ancFN3KRP R191D Forward Primer	CGAGAAAGACTATGGGGACCGCGAGGCC
ancFN3KRP R191D Reverse Primer	CCATAGTCTTTCTCGATCATGTCCAGTTGGGG
ancFN3KRP Y253F Forward Primer	TTCCGAGTTTGAGCTGGCTATCGCGGGAATG
ancFN3KRP Y253F Reverse Primer	AGCTCAAACTCGGAATGGCCGTAGAAACTTG
ancFN3KRP Y269F Forward Primer	GTCCTTCTTCTCAGCTTACCACAGCAAAATTCCG
ancFN3KRP Y269F Reverse Primer	GCTGAGAAGAAGGACGAGGAGAATCCGCC
ancFN3KRP H293N Forward Primer	ATTATTTAACTACCTTAACCATTGGAATCATTTCG
ancFN3KRP H293N Reverse Primer	AGGTAGTTAAATAATTGGTACAATTTTAAACGC
ancFN3KRP N315K Forward Primer	TATGCGTAAATTGTTGAAGTGCTTAAAAGCCTAG
ancFN3KRP N315K Reverse Primer	AACAATTTACGCATAATATTAAGACTACTGGAG
ancFN3K N74E Forward Primer	GAAAACAGAAACCGTAAAAGTACCGAAGCC
ancFN3K N74E Reverse Primer	ACGGTTTCTGTTTTCAGAATGGCCTCCAGC
ancFN3K P145V Forward Primer	ATCGGAAGTACAATATGTGGATAAATTCGGTTTTC
ancFN3K P145V Reverse Primer	TATTGTACTTCCGATTGACCAGCACCC
ancFN3K I162L Forward Primer	TGGATACTTACCTCAAGTGAACGAGTGGC
ancFN3K I162L Reverse Primer	TGAGGTAAGTATCCACAGCAAGTCACTG
ancFN3K P173V Forward Primer	TGACTGGGTGACCTTTTTCGCGCGTCACC
ancFN3K P173V Reverse Primer	AAGGTCACCCAGTCATCTTGCCACTCG
ancFN3K H179Q Forward Primer	CGCGCGTCAACGCTTGCAAGCGCAGCTTG
ancFN3K H179Q Reverse Primer	AAGCGTTGACGCGCGAAAAAGGTCGG
ancFN3K A183P Forward Primer	CTTGCAACCTCAGCTTGACTTAATTGAGAAGGAC
ancFN3K A183P Reverse Primer	AGCTGAGGTTGCAAGCGGTGACGCGC
ancFN3K L187M Forward Primer	GCTTGACATGATTGAGAAGGACTATGGTGACCGC
ancFN3K L187M Reverse Primer	TCAATCATGTCAAGCTGCGCTTGCAAGC

ancFN3K D191R Forward Primer	TGAGAAGCGTTATGGTGACCGCGAAGCACG
ancFN3K D191R Reverse Primer	CCATAACGCTTCTCAATTAAGTCAAGCTGCGC
ancFN3K F253Y Forward Primer	CTCGGAGTATGAATTAGCGATCGCTGGTATGTTTG
ancFN3K F253Y Reverse Primer	AATTCATACTCCGAGTGTCCATAGAAGGATGC
ancFN3K F269Y Forward Primer	TAGTTTTTATTCTGCATACCATTCAAAGATCCC
ancFN3K F269Y Reverse Primer	GCAGAATAAAAACTACTGCTAAAGCCCCC
ancFN3K N293H Forward Primer	ACTTTTCCATTACCTTAATCATTGGAACCATTTTG
ancFN3K N293H Reverse Primer	AGGTAATGGAAAAGTTGGTATAATTTCAAACGC
ancFN3K K315N Forward Primer	TATGCGTAATTTGCTTAAATGTTTGAAAGCGTAGA
ancFN3K K315N Reverse Primer	AGCAAATTACGCATAATGTTAAGCGAACTACT
HsFN3K I25A forward	AGGGTGTGCGAGCGAAGGCCGTGCTTACG
HsFN3K I25A reverse	TCGCTCGCACACCCTGCGCCGGGACC
HsFN3K D217A forward	CATGGCGCGCTTTGGTCGGGAAATGTG
HsFN3K D217A reverse	CAATAATGCAGGTACGATCTCCAG
HsFN3K W219A forward	CATGGCGATCTTGCGTCGGGAAATGTG
HsFN3K W219A reverse	CAATAATGCAGGTACGATCTCCAG
HsFN3K F252A forward	TTTAATGGCGGGTGGGTTTCCCCGTAGTTTTTC
HsFN3K F252A reverse	CCACCCGCCATTAAAGCGATAGCAAGTTCAAAC
HsFN3K N287A forward	TTACTTAGCGCATTGGAACCATTTCGGTCGTG
HsFN3K N287A reverse	CAATGCGCTAAGTAATTAAAAAGTTGATACAG
HsFN3K H288A forward	TACTTAAACGCTTGGAACCATTTCGGT
HsFN3K H288A reverse	ATTAAAAAGTTGATACAGAAGCAGACG
HsFN3K H291A forward	AACCATTGGAACGCTTTCGGTCGTGAAT
HsFN3K H291A reverse	TAAGTAATTAAAAAGTTGATACAGAAG
HsFN3K F292A forward	AAACCATTGGAACCATGCCGGTCGTGAAT
HsFN3K F292A reverse	TAAGTAATTAAAAAGTTGATACAGAAG
HsKT3K forward	GGCACGTAGGATCCATGGAAGAGTTATTACGTCGC
HsKT3K reverse	AAGTCAATAAGCTTCTAGTGGTGGTGGTGGTGGTGCTT CACAAG

Supplementary Table 2. A list of primers used in the generation of expression plasmids of ancFN3Ks and HsFN3K.
