# Supplementary material for: Ancestral protein reconstruction reveals the mechanism of substrate specificity in FN3K-mediated deglycation
Source: Commun Biol. 2026 Apr 3;9:738. doi: 10.1038/s42003-026-09967-3 (PMC13223202; doi:10.1038/s42003-026-09967-3)
Supplement: Supplementary file 2 — Description of Additional Supplementary Materials [file 42003_2026_9967_MOESM2_ESM.pdf]

Description of Additional Supplementary Files

File name: Supplementary Data

Description: Experimental data generated in this study
